# Development and evaluation of X‐ray lithophanes as test objects for interventional procedure simulation

**DOI:** 10.1002/mp.70742

**Published:** 2026-10-28

**Authors:** Carlo Saija, Madeleine Dietrich, Federica Facente, Wen Wei, Campbell Littlefield, Mariia Kryshchuk, Pablo Aparicio‐Villar, Nassim Boudjatit, Antonia Pontiki, Richard James Housden, Eric Sejor, Kawal Rhode, Pierre Berthet‐Rayne

**Affiliations:** ^1^ School of Biomedical Engineering & Imaging Sciences King's College London England UK; ^2^ Caranx Medical, Nice Provence‐Alpes‐Côte d'Azur France; ^3^ Department of Biomedical Engineering Case Western Reserve University Cleveland Ohio USA; ^4^ Inria Centre Université Côte d'Azur, Valbonne Provence‐Alpes‐Côte d'Azur France; ^5^ Department of Psychology and Neuroscience University of North Carolina, Chapel Hill North Carolina USA

**Keywords:** artificial intelligence, fluoroscopy, lithophane, patient replica, phantom, simulation

## Abstract

**Background:**

In the growing field of interventional radiology, emerging technologies implement the use of artificial intelligence (AI) to help tackle growing demands for treatment. Due to the anatomical differences in animals, volunteers offer the most realistic training and testing scenario, however safety considerations restrict rapid prototyping of these new technologies. Simulators have been a key facilitator in the testing of new technologies among numerous medical engineering studies, but often lack the realism to reliably test AI.

**Purpose:**

This proof‐of‐concept study presents methods to create or enhance hyper‐realistic radiographic simulators that can effectively function with a medical‐grade AI software.

**Methods:**

Iron‐filled plastic was used to three‐dimensionally (3D) print a series of blocks of heights up to 30 mm. By analyzing the intensities generated by different thicknesses of plastic, anonymous fluoroscopic images were turned into height maps (lithophanes) and 3D‐printed. A static scene from transcatheter aortic valve implantation (TAVI) was then 3D‐printed and imaged with different presets using two different x‐ray systems for mathematical analysis. Lithophanes were then used as a radiographic background to experimental and commercial interventional simulators to test Caranx Medical's TAVIPILOT AI. Finally, fluoroscopies from 5 3D‐printed lithophanes were mixed with 5 real images and presented to 5 expert volunteers to establish their realism in a structured survey.

**Results:**

The 3D‐printed x‐ray lithophanes test‐plates' radiographic appearance was rated as “Adequate” to “Good” across all fields of the survey, with participants praising their realism. The test‐plate was able to reliably recreate the TAVI scene in different x‐ray systems, with joint‐histogram correlation coefficients above 0.9, and global structural similarity above 0.8. The lithophanes were imaged as a standalone test plate and as a backing to dynamic TAVI interventional simulators; in both cases, TAVIPILOT AI responded to the simulator as if it was operating on a real anatomical image.

**Conclusions:**

Lithophane plates present an easy‐to‐implement solution to recreate monoplane x‐ray scenes, which can be deployed to test software at the point of care with minimal logistical, ethical, or safety concerns. This study opens the door for a new range of interventional simulators able to recreate human‐like appearance to test AI, offering a non‐exclusive alternative to patient volunteers and anonymous data.

## 1 INTRODUCTION

The growing adoption of minimally invasive techniques and preventative care^1^, ^2^ has driven rapid prototyping in radiology software and surgical robotic systems aimed at improving procedural guidance and optimizing patient safety^3^; however this research must be accelerated in ways that preserve patient safety and ethics.^4^

Fluoroscopy, a form of non‐invasive x‐ray imaging technique, employs a pulsed x‐ray beam with a flat‐panel detector to create two‐dimensional grey‐scale image projections of the patient's anatomy over time, effectively generating a live video for operator guidance.^5^ Due to the nature of the detector array, the greater the number of x‐ray photons reaching the detector, the greater the intensity value of the image.^6^ Each fluoroscopy system employs a series of filters, such as normalization,^7^, ^8^ to maximize image quality and signal‐to‐noise ratio, ensuring all features are clearly visible. To test and calibrate these parameters, artificial test objects and phantoms are routinely used as a repeatable standardized test scenario.

In the expanding field of fluoroscopy‐guided valve replacements and implantations,^9^, ^10^ Transcatheter Aortic Valve Implantation (TAVI) is the leading intervention addressing the rising demand for aortic valve stenosis treatment, the most common valvular heart disease in developed countries.^11^, ^12^ During the procedure, operators place a diagnostic pigtail catheter in the non‐coronary cusp and a stiff preformed guide‐wire inside the left ventricle (Safari 2, Boston Scientific, Marlborough, USA).^13^ Iodinated contrast agent is injected into the aortic root using the diagnostic catheter, allowing operators to identify the cusps of the valve and align the fluoroscopy x‐ray systems orthogonally to the valve annulus, providing a more standardized view of the area of interest.^13^ Under fluoroscopic guidance, operators use a steerable balloon catheter device to position the balloon‐expandable implant. The preformed stiff guide‐wire assists in navigating the catheter into place.^10^ The operators align the implant with the previously identified native valve annulus and expand it into place with the balloon of the delivery device. To meet the growing demand for interventional imaging and optimize procedural outcomes, new technologies, such as AI and robotics, have been proven to enhance diagnostic outcomes, provide real‐time guidance with live tracking, and improve intravascular image analysis.^14^

The TAVIPILOT software (Caranx Medical, Nice, France) uses AI to detect key anatomical landmarks and instruments during TAVI in live fluoroscopy, specifically identifying the pigtail catheter, the transcatheter aortic valve, and the aortic cusps. TAVIPILOT has been cleared (510(k)) by the Food and Drug Administration (FDA) to guide clinicians in real‐time during TAVI procedures using live fluoroscopy and augmented reality, with the primary goal of guiding clinicians during prosthetic valve implantation.^15^, ^16^ The AI system was developed using data from patient images acquired during TAVI procedures, ensuring its adaptability across diverse clinical scenarios and patient anatomies.^15^, ^16^ A new experimental (non cleared) version of TAVIPILOT integrates with a robotic platform^17^ that assists in precisely positioning of the valve delivery system, aiming to provide enhanced control and reduce the potential for human error. At the time of this study, this new version of TAVIPILOT is still under experimentation, thus providing the perfect opportunity to test new simulation technologies in the context of AI‐robotics testing.^17^

Conventionally, tests in interventional techniques, medical imaging, and surgical practice are often performed on live pigs, dogs, or sheep (2250‐2750€  for acute tests),^18^, ^19^, ^20^ or fresh revitalized cadavers (1250‐1550€).^21^, ^22^ This however presents complications due to the additional logistics, costs, and shortage of donated cadavers. Phantoms and simulators have played an increasingly crucial role in testing new medical technologies and training new staff.^23^, ^24^ In radiology, plastics and rubbers are often implemented for simulating body tissues due to their similar x‐ray attenuation.^25^ N. Irnstorfer et al.^26^ utilized polylactic acid (PLA) plastic and 3D‐printing resin (polyacrylate) to create x‐ray lithophane‐like test objects for neonatal pediatrics (see Figure 1). The study analyzed thicknesses up to 7.5 cm of plastic to replicate a wide range of intensities in the image.^26^ Using relatively low energy x‐ray (66 pkV), their team was able to recreate the appearance of neonatal chests using lithophane plates with similar thickness to the original patients. Though effective for small newborns, the methods and material choice adopted by N. Irnstorfer et al.^26^ would arguably present limitations when reproducing images acquired from larger adults at higher x‐ray energy values. To effectively replicate most uniplanar x‐ray images, the lithophane concept should be implemented using a more attenuative materials, attenuating more x‐ray photons with less thickness.

**FIGURE 1:**
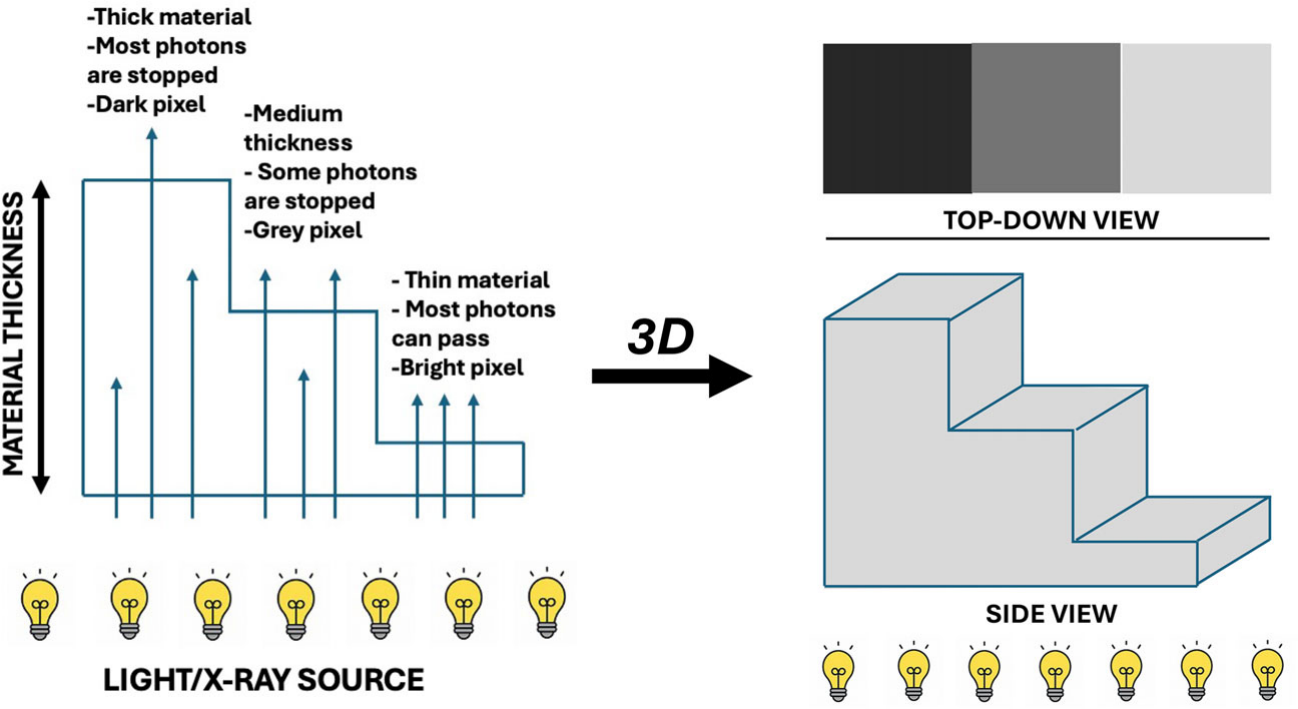
This illustration depicts the working principle of lithophanes. When shining light/x‐ray from below though an opaque/radiopaque object and looking from a top‐down perspective, the intensity (brightness) of each pixel is inversely proportional to the material thickness. Applying this to a larger array of pixels allows users to reproduce a full grey‐scale image.

Similarly to imaging, simulators have recently found growing use in the testing of cardiovascular interventional technologies, facilitating prototyping, and standardized testing.^23^, ^24^, ^27^, ^28^, ^29^, ^30^ Thin cardiovascular rubber/acrylic phantoms have been used either in water or in air to test and evaluate the performance of catheter robots^27^ and tool tracking deep learning software^28^, ^29^, ^30^ under fluoroscopic guidance. Unlike in‐vivo or ex‐vivo models, the physical simulators provided an ethical, controlled, and replicable scenario, without having to expose volunteers to radiation.

With new more sophisticated AI‐powered radiology software and x‐ray guided robotics, conventional test scenarios are often not realistic, repeatable, or numerous enough to reliably and safely test new systems and protocols.^31^, ^32^ Due to fundamental anatomical and radiographic differences between humans and animals or phantoms, AI systems such as TAVIPILOT, which are trained exclusively on human imaging data cannot be reliably evaluated using non‐human datasets. Annotating or training on animal or phantom fluoroscopy would yield a model applicable only to animal or phantom anatomy and thus be irrelevant for assessing human safety or clinical performance. Most physical interventional simulators on the market^33^, ^34^ and in literature^34^, ^35^ only focus on replicating the anatomy of interest and its physical properties, as replicating all anatomical features in x‐ray would prove impractical, complex, and/or costly.^36^ Augmented reality and virtual reality simulations would involve manipulating images before providing them to the software,^37^ rendering experiments in the cardiac catheterization laboratory less realistic or impractical.^37^, ^38^, ^39^ This fuels the development of new more sophisticated simulators, which can reliably replicate human‐like properties without needlessly exposing volunteers or staff to additional radiation and repeated tests.

To tackle the need of hyper‐realistic human‐like radiographic simulators, this research study presents a novel method to 3D‐print custom x‐ray patient replicas in the form of thin Fe‐PLA lithophanes, which can be directly imaged in the catheterization laboratory. The manuscript describes three proof‐of‐concept simulations: (1) recreating a single fluoroscopy frame acquired during a TAVI procedure, (2) dynamically simulating implant placement by overlaying a lithophane with a rigid 3D‐printed aortic phantom, and (3) using lithophanes as a background for a more advanced, commercially available flexible aortic phantom. Finally, the lithophanes' realism was evaluated mathematically using Matlab, by TAVIPILOT AI, and by five human observers in a dual‐stage blind survey with structured Likert scale feedback. The use of lithophanes in this study introduces real human data to physical phantom models, achieving levels of realism only comparable to advanced virtual reality such as the Angio Mentor (Surgical Science, Göteborg, Sweden) and other similar systems.^40^ This low‐cost method opens possibilities to test AI and other systems on replicated human data without involving animals, human subjects, cadavers, or integrating virtual reality into the catheterization laboratory.

## 2 METHODS

### 2.1 Material choice

In radiology, the ability of x‐ray photons to pass through an object and reach the detector is dictated by the Lambert‐Beer law:

(1) $$I=I_{0}\cdot e^{-\mu x}$$

where I is the intensity of the beam, $I_{0}$ is the original intensity of the incident beam, μ is the linear attenuation coefficient of the medium, and x is the length of the path taken.^41^ The attenuative properties of a material in x‐ray imaging are mostly determined by the energy of the beam (pkV), the atomic number of its constituent atoms, and their distribution within the material (density). Materials with higher atomic numbers and densities are more likely to interact with the x‐ray photons, thus making them more attenuative.^41^, ^42^ Linear attenuation coefficient is the physical property which quantifies this phenomenon, and Hounsfield units (CT number) compare these coefficients to that of water.^42^ At 120 kV, PLA has a CT number of 183±8HU,^25^ while steel has a CT number of 20,346±63.6HU^43^; body tissues in the chest including adipose, blood, muscle, and bone have CT numbers ranging from ‐89HU to 1500HU.^44^, ^45^ In the case of fluoroscopy, thicker sections of radiopaque materials result in more interactions as the photons travel through longer attenuating paths (x), making the object appear darker.

The x‐ray lithophanes presented in N. Irnstorfer et al.^26^ were limited to reproducing neonates and small body parts due to their material choice of PLA and acrylic resin, which are hydrocarbons. Iron‐filled PLA (Fe‐PLA) (Protopasta, Vancouver, USA) is a 3D‐printing plastic containing iron powder. With a density of 1.85 $gc^{-3}m$, the material is composed of 9.2% iron powder and 90.8% PLA by mass.^46^ This composition gives the material a higher CT number than standard PLA (2449 ±45 HU at 120 kV), enabling it to attenuate more x‐ray photons with less thickness.^25^ This plastic has previously found application in simulating bone tissue for CT^25^ using infill patterns to replicate the spongy bone and the outer walls to replicate cortical bone.

### 2.2 Mathematical modelling

In order to determine the range of achievable 8‐bit pixel intensities and the thickness necessary to achieve them, a series of 2 x 2 cm Fe‐PLA blocks was 3D‐printed with thicknesses: 1, 3, 6, 9, 12, 15, 18, 21, 24, 27, and 30 mm. A Siemens Artis Q x‐ray system (Siemens, Berlin, Germany) was oriented in PA (posterior‐anterior) view and set to image using a high dose cardiac preset protocol (Protocol 1) (81 pkV, 0.140 mA, 15 Hz framerate). Blocks were incrementally added into the field of view as shown below in Figure 2.

**FIGURE 2:**
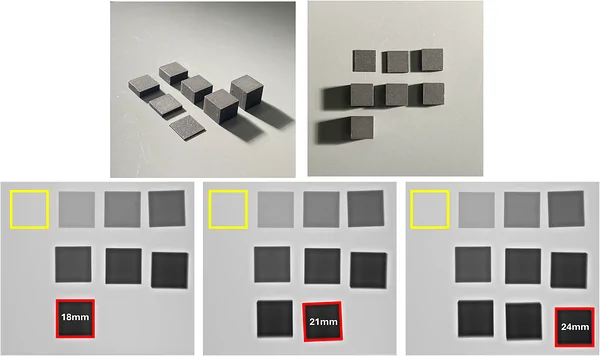
This illustration shows how the darkest (minimum) intensity was acquired from the tallest block (1–30 mm) in the image (marked in red), and how the brightest (maximum) intensity was acquired from the background of the image.

The fluoroscopy sequences were exported as 8‐bit DICOM images, and the average pixel intensity and standard deviation of the thickest block was measured with ImageJ (ImageJ, Hayward, USA). Similarly, an equivalent section of the background (2 x 2 cm) was sampled for each image as well. The thickest block and the background provide an estimate of the darkest and brightest pixels in the lithophane. Pixel intensities and their range may vary based on the x‐ray system and its setting. However, thicker sections of material should stop more photons, resulting in darker pixel intensities. These darker areas tend towards the minimum pixel value defined by the system settings. The blocks' pixel intensity versus maximum thickness (min. intensity vs. thickness) was therefore plotted in MATLAB (MathWorks, Natick, USA) and a line of best fit was iteratively derived (model 1) based on the decay Equation (2):

(2) $$I_{\min}=a\times e^{-b(T-c)}+d$$

where a, b, c, and d are scalar constants to be estimated. To optimize contrast, x‐ray systems and imaging settings can make the non‐attenuative background appear brighter than the radiopaque objects. To account for this effect, the background intensity (max. intensity vs. thickness) (model 2) was linearly plotted on MATLAB as shown in Equation (3):

(3) $$I_{\max}=a\times T+b$$

where a and b are scalar constants to be estimated. The variable T represents the thickness of the tallest block in the image and variable I_max_ represents the mean intensity of that block. The coefficients for model 1 and model 2 were iteratively derived using MATLAB inbuilt functions. The mathematical functions provided a map of the minimum and maximum intensity that could be achieved with these x‐ray settings using the Fe‐PLA lithophanes of thicknesses 1–30 mm.

### 2.3 Lithophane generation

MATLAB code was used to turn images into 3D triangular meshes (Standard Triangle Language files, STL). The user inputs the desired image and the dimensions they would like. Next, the input image is turned into an 8‐bit integer array. The darkest pixel intensity (min. intensity) of the desired image was measured to derive the necessary lithophane thickness using mathematical model 1. This maximum thickness was then plotted against mathematical model 2 to estimate the brightest achievable intensity (max. intensity) for that lithophane. The 8‐bit array was then edited: all pixel values higher than or equal to max. intensity were capped to equal max. intensity. The final array was then normalized into a linear height map. Finally, the heigh map is proportionately enlarged based on the input dimension (without cropping) and saved as an STL file ready for 3D printing. All lithophanes were restricted to be at least 0.8 mm thick and no more than 30 mm thick to maintain structural integrity and avoid material waste. This process of lithophane generation is graphically shown in Figure 3.

**FIGURE 3:**
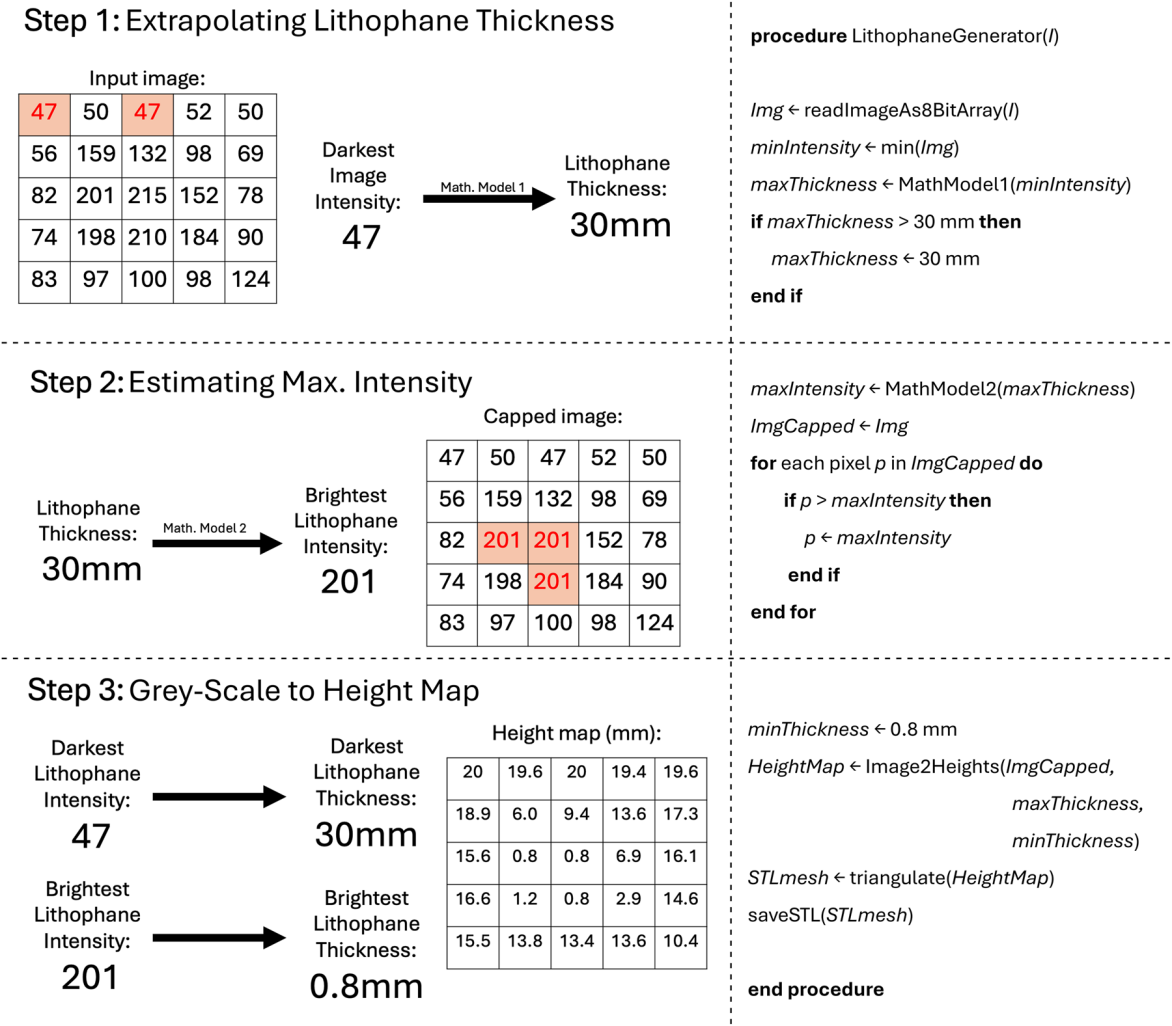
Step 1: The darkest pixel in the image is used establish the thickness of the lithophane using mathematical model 1. Step 2: mathematical model 2 uses the lithophane thickness to calculate the brightest possible intensity achievable with the lithophane (max. intensity). All higher pixel values in the image are then capped to the max. intensity value. Step 3: all pixel values are normalized and turned into a height map from 0.8 mm to the previously calculated lithophane thickness.

### 2.4 Simulation 1 ‐ static lithophane image

As a proof of concept, one fluoroscopy sequence was acquired from anonymized TAVI patient data using the GE ODEC Elite x‐ray system (GE Healthcare, Chicago, USA) (76.8 pkV, 0.564 mA, 15 Hz framerate). The image shows the patient anatomy and the TAVI equipment during the valve deployment. The image was turned into a 15 x 15 cm STL (Figure 4a) file using the MATLAB code. The lithophane was 3D‐printed in Protopasta Fe‐PLA at 0.2 mm layer height with 100% infill, using a 0.4 mm hardened steel nozzle on the Bambu Lab X1‐Carbon 3D‐printer (Bambu Lab, Shenzhen, China) (Figure 4b). The lithophane was imaged using the Siemens Artis Q x‐ray system with Protocol 1 and another high‐dose cardiac preset protocol (Protocol 2) (81 kV, 0.120 mA, 15 Hz framerate). Similarly to patient practice, magnification and collimation were optimized to center the area of interest of the lithophane. The same experiment was repeated using a fluoroscopy high dose preset protocol (Protocol 3)(80 keV, 0.040 mA, 15 Hz framerate) on the Siemens Cios Spin x‐ray system (Figure 4c,d).

**FIGURE 4:**
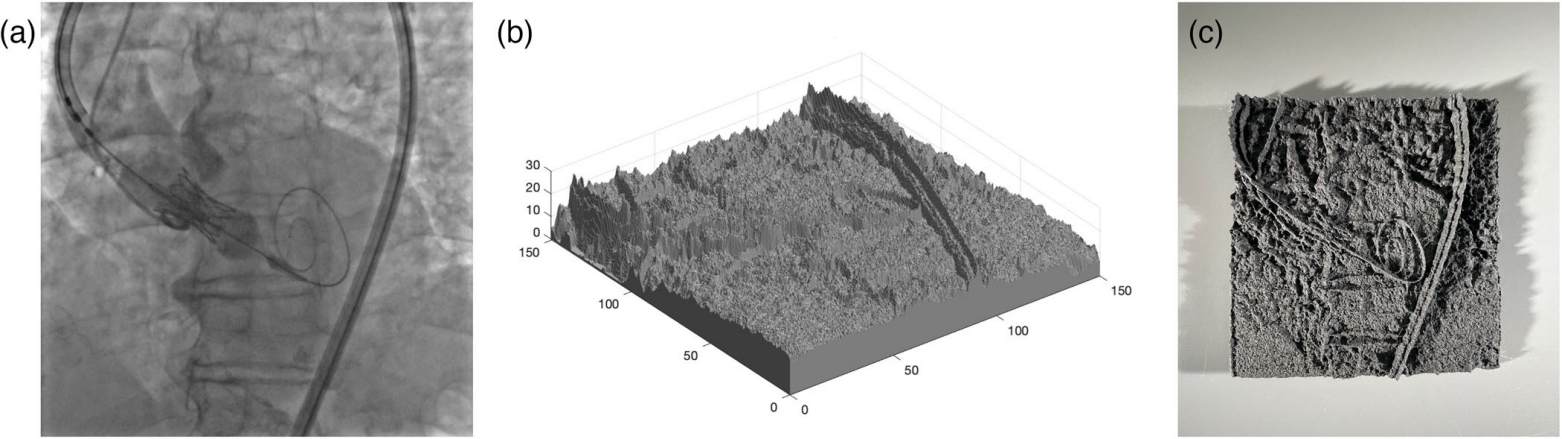
(a) The original anonymous patient TAVI image used to generate the lithophane for Simulation 1. This image was captured mid‐way through the process of implant deployment, when the balloon is half‐inflated. (b) The STL file generated on MATLAB for Simulation 1. (c) The 3D‐printed x‐ray lithophane for Simulation 1 placed on the bed of the Siemens Artis Q x‐ray system.

### 2.5 Simulation 2 ‐ lithophane integration into conventional rigid phantom

In this second proof‐of‐concept simulation, the lithophane was paired with a physical TAVI simulator. The simulator was composed of an acrylic aortic phantom with a rubber aortic valve, a base to hold it, and a tube to introduce catheters. A tool‐free fluoroscopy image was obtained from anonymized TAVI patient data and turned into a 3D‐printed lithophane as described in Section 2.3. The shape of the aortic phantom was custom made to complement the anatomy of this patient, so the aortic phantom could be placed in the simulator base to overlay the lithophane and replicate patient anatomy. The fully assembled simulator (Figure 5a,b,d) was imaged using the Artis Q x‐ray system with Protocol 1 as a 316 stainless steel 3D‐printed phantom of the Sapien 3 implant (Edwards Lifesciences, Irvine, USA) (Figure 5c) was being placed and deployed using a delivery catheter (Edwards Commander (Edwards Lifesciences, Irvine, USA)) (Figure 5).

**FIGURE 5:**
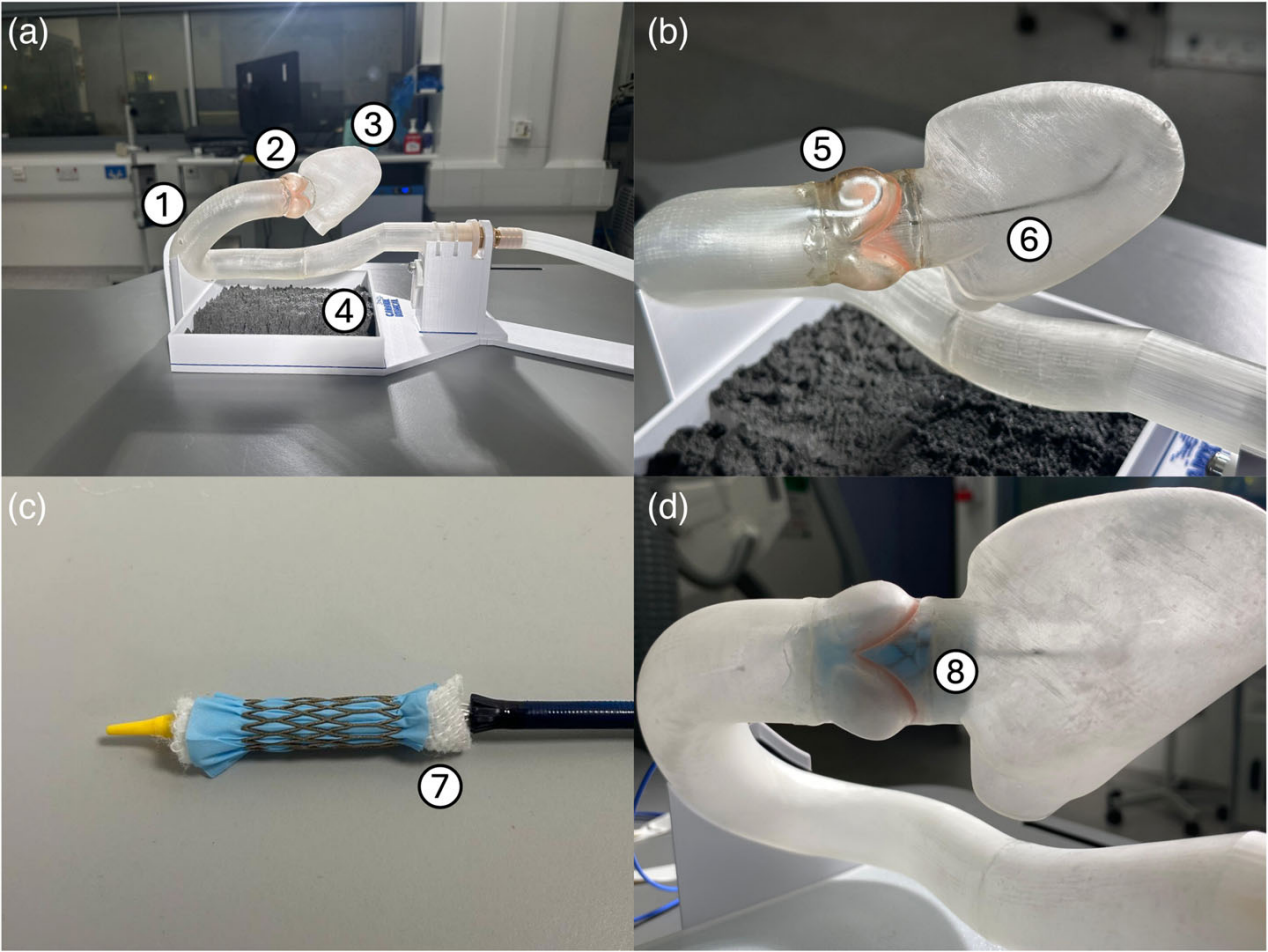
(a) In Simulation 2, the acrylic aorta (1), with its aortic valve (2) and left ventricle (3), was overlaid with the tool‐free lithophane (4) to provide a more realistic background appearance. (b) The pigtail catheter (5) and the Safari guide‐wire (6) were placed in the non‐coronary cusp and the left ventricle respectively. (c) The 3D‐printed stainless steel Sapien3 stent phantom (7) was designed based on publicly available information.^47^ D: The stent (7) was crimped onto the Edwards balloon catheter and deployed (8) in the aortic phantom by inflating the balloon with water.

### 2.6 Simulation 3 ‐ lithophane integration into flexible beating phantom

In this third and final proof‐of‐concept simulation, the lithophane was paired with Pangolin Medical's silicone beating heart phantom (Pangolin Medical, En Carmel, Israel) placed inside of a tank of water. Using the same tool‐free lithophane background as Simulation 2, the shapes of the aortic phantoms were aligned to complement the anatomy of the lithophane. The beating Pangolin simulator (Figure 6) was imaged using the Artis Q x‐ray system using a high dose cardiovascular preset protocol (Protocol 4) (125 kV, 0.208 mA, 7.5 Hz framerate) as a 316 stainless steel 3D‐printed phantom of the Meril Octapro implant (Meril Life Sciences, Vapi, India) was being placed and deployed using the Meril delivery catheter (Figure 6). To further test the capabilities of lithophane‐enhanced simulators, the new (non cleared) version of TAVIPILOT was used to detect landmarks live, whilst the AI drove the Caranx Medical robotic system to align the implant with the aortic valve.

**FIGURE 6:**
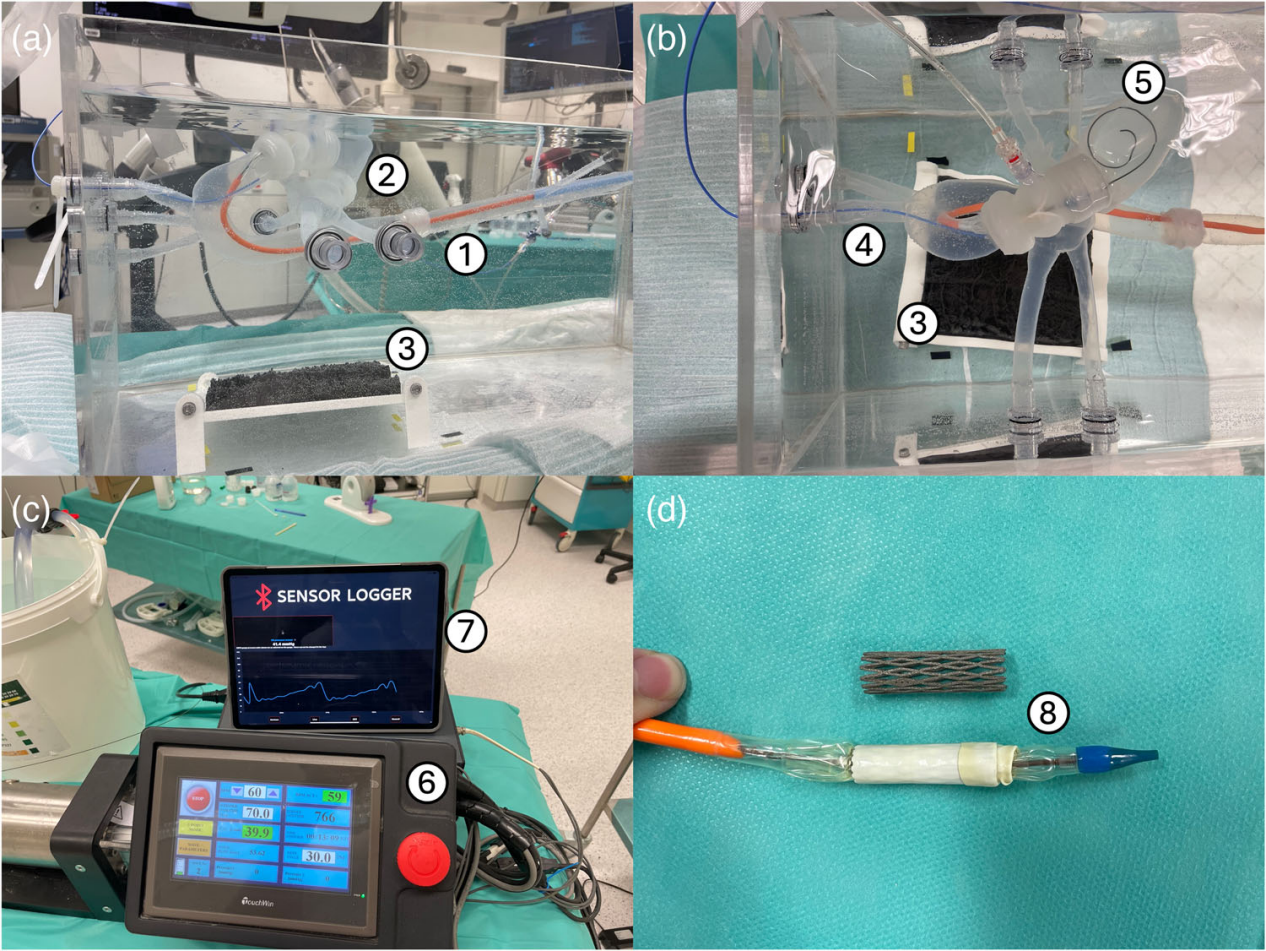
(a) During Simulation 3, the Meril balloon delivery catheter (1) was navigated inside the Pangolin Medical silicone rubber simulator (2) in a water tank, with the lithophane (3) as a background. (b) Line in Simulation 2, the pigtail catheter (4) and Safari guide‐wire (5) were positioned in the aortic valve and the ventricle. (c) The phantom was perfused using the simulator's pump (6) monitoring flow settings (7) controlled throughout the tests. (d) A phantom of the Meril Octapro implant (8) was reconstructed based on designs by Kilik T. et al.^47^ and 3D‐printed in 316 stainless steel.

### 2.7 Mathematical evaluation

In order to quantitatively assess the lithophane's ability to recreate the original image, the three lithophane images acquired from Simulation 1 were compared to their original patient fluoroscopy image. The three pairs of images (patient vs lithophane) were automatically aligned and cropped using inbuilt functions “detectSURFFeatures,” “extractFeatures” and “estimateGeometricTransform” on Matlab. Using Matlab inbuilt functions, the pairs were evaluated based on: the 2D joint histogram and 2D correlation coefficient, normalized 8‐bit root mean squared error (NRMSE), an intensity difference map, mean intensity difference, and structural similarity index (SSI) (global and local). Image sharpness was quantified using the Variance of Laplacian (VoL).

### 2.8 AI‐based evaluation

To test the lithophanes as a human‐like test environment for AI and software prototyping, all x‐ray images form Simulation 1 and Simulation 2 were submitted post‐operatively to the FDA approved version of TAVIPILOT for analysis. The program was set identify the non‐coronary cusp of the aortic valve, and mark the pigtail catheter and the four vertices of the implant and mark them with colored dots on the image, highlighting all landmarks during the alignment/deployment phase with a particular focus on the valve.

### 2.9 Survey‐based evaluation

To test the validity of lithophanes as a simulation tool from a human perspective, five lithophanes were crafted and imaged using the Artis Q x‐ray system with Protocol 1. They displayed five instances of TAVI: an overview of the chest during guide‐wire placement, implant alignment, implant deployment, a view of the implant post‐deployment, and tool extraction at the hip. Five other images from different anonymous patients were selected and mixed with the lithophane images. The evaluation was structured as a two‐part survey. In the first part, participants were shown randomized pairs of images as follows: lithophane‐lithophane, real‐real, lithophane‐real, real‐lithophane (20 pairs in total). Pairs were randomly shuffled as to remove repeating patterns and thus minimize bias. As they were shown the pairs, participants were asked to answer “Which image looks realistic?” and could select “Left,” “Right,” “Both,” or “Neither.” This was performed to assess the participant's proficiency in recognizing fakes. In the second part of the survey, participants were asked to rate the lithophane images based on a 5 point Likert scale in the fields of: “Image Sharpness,” “Fidelity to Clinical Appearance,” “Grey‐Scale Contrast,” “Noise Management,” “Visibility of Critical Details,” “Overall Realism/Diagnostic Usability.” The scale ranged from “Very Poor (1),” to “Adequate (3),” to “Excellent (5).” Participants were recruited in the research catheterization laboratories at IHU De Strasbourg; the population included clinical staff and AI software engineers working on fluoroscopy research. At the time of the study, all volunteers see fluoroscopy data weekly, with two reviewing four or more cases a day. Their years of experience varied from two years in their current role to thirty years. During the entirety of the test participants were not made aware of which images were real and which were lithophanes. To maintain the volunteers' anonymity, participants were provided a full description of the study and understood that answering the questionnaire implied consent, so no traceable information would be collected (MRSP‐24/25‐51316). The images were displayed in a darkened control room (<50 lux ambient lighting^48^) on the Karl Storz 200905 19 radiographic screen (Karl Storz SE & Co., Tuttlingen, Germany) positioned at eye level, with a viewing distance of 30–70 cm.^49^

## 3 RESULTS

### 3.1 Lithophane generation

In the range of thicknesses analyzed, mathematical model 1 (shown in Figure 7 on the left) follows an exponential decay where the scalar values a, b, c, d were estimated to be 117.2, 0.1016 ${\text{mm}}^{-1}$, 1 mm, and 30.75 respectively. The line of best fit closely follows all data points on the graph. 2D cross correlation between the line of best fit and the data points in model 1 was 0.933 (*p* < 0.0001).

**FIGURE 7:**
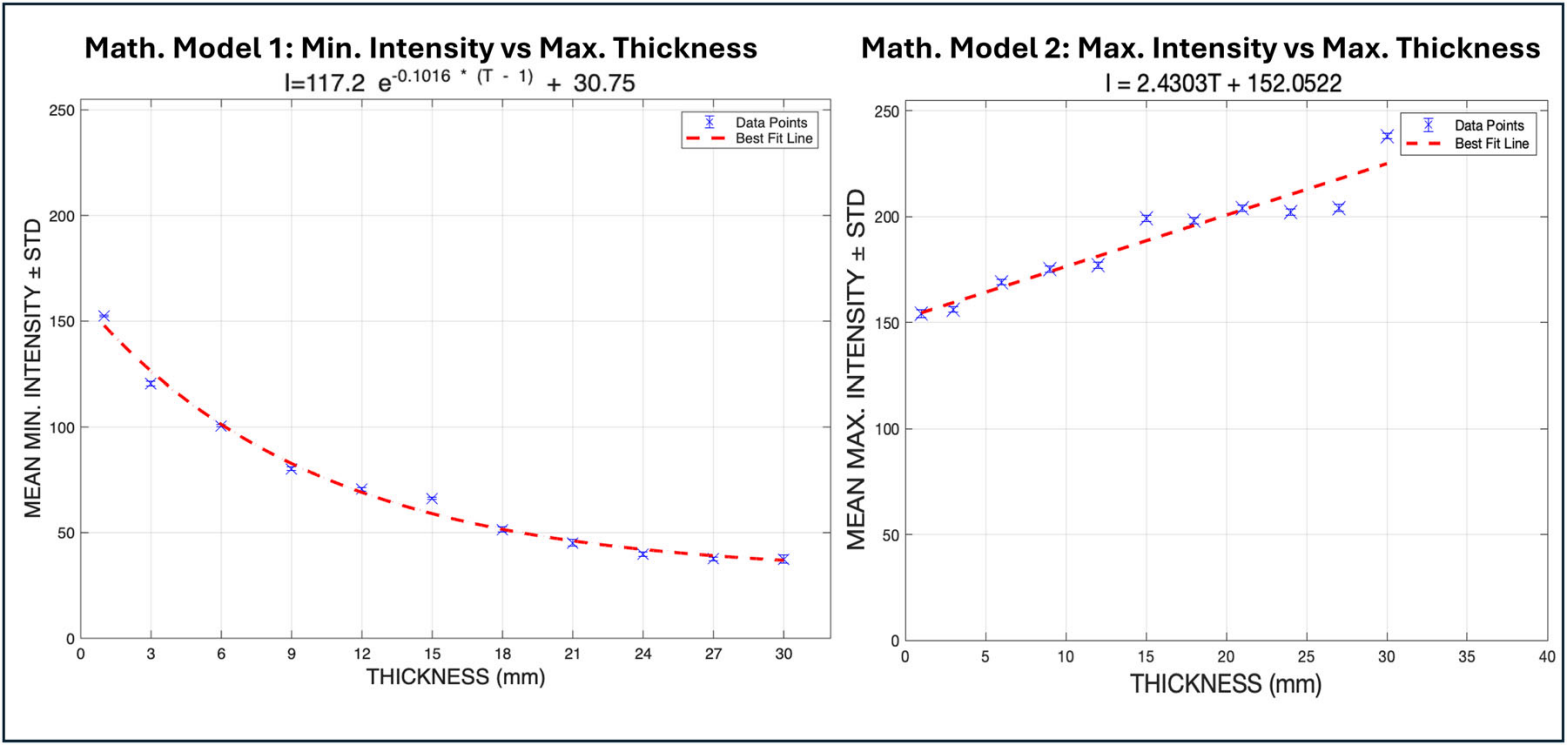
The graph on the left and on the right depict mathematical models 1 and two respectively. The red dotted lines mark the lines of best fit and their equations are noted as subtitles on each graph.

Mathematical model 2 (shown in Figure 7 on the right) was linear, with coefficients a and b equating to 2.4303 ${\mathrm{mm}}^{-1}$ and 152.0522 respectively. The linear correlation coefficients for the points recorded in this graph was 0.953 (*p* < 0.0001).

The lithophane for Simulation 1 was 3 cm thick; it was 3D‐printed over a time of 14 h and 8 min. The material cost for this model was of £64.83. The lithophane for Simulations 2 and 3 was 2.54 cm thick; it was 3D‐printed over a time of 14 h and 37 min. The material cost for this model was of £56.14.

### 3.2 Mathematical evaluation

Figure 8a,b displays the results achieved with the Artis Q x‐ray system. With Protocol 1 (Figure 8a), the peak energy achieved for this sequence was 77.1 pkV. The joint histogram follows a linear pattern (Figure 8), with a 2D correlation coefficient of 0.839. The NRMSE between the two images was 0.0656. The cropped original image displayed an average intensity of 118±23, while the cropped lithophane image showed an average intensity of 128±27. The mean pixel difference between the two images was of ‐10±14, with the most noticeable differences taking place around the catheter guide‐wires and tools. The global SSI was 0.882, with the most noticeable differences taking place around the Safari guide‐wire, catheter, and implant. With Protocol 2 (Figure 8b), the peak energy achieved for this sequence was 74.6 pkV. The joint histogram follows a linear pattern (Figure 8), with a 2D correlation coefficient of 0.933. The NRMSE between the two images was 0.0393. The cropped original image displayed an average intensity of 119±23, while the cropped lithophane image showed an average intensity of 120±29. The mean pixel difference between the two images was of ‐1±10, with the most noticeable differences taking place around the catheter guide‐wires and tools. The global SSI was 0.827, with the most noticeable differences taking place around the Safari guide‐wire, catheter, and implant. Figure 8c displays the results achieved with the Cios Spin x‐ray system. The peak energy achieved for this sequence was 80.0 pkV. The joint histogram follows a linear pattern (Figure 8), with a 2D correlation coefficient of 0.946. The NRMSE between the two images was 0.0599. The original image displayed an average intensity of 120±24, while the lithophane image showed an average intensity of 128±34. The mean pixel difference between the two images was of ‐9±12, with the most noticeable differences taking place around the catheter, guide‐wires, and spine. The global SSI was 0.623, with the most noticeable differences taking place in the aortic root on the guide‐wire and catheter. The image displays some speckle noise, which can also be noticed on the local SSI map. The VoL reveals that the original patient image (VoL = 37) was sharper than the images achieved Protocols 1 and 2 (VoL = 2 and VoL = 21 respectively), but not as sharp as Protocol 3 (VoL = 354).

**FIGURE 8:**
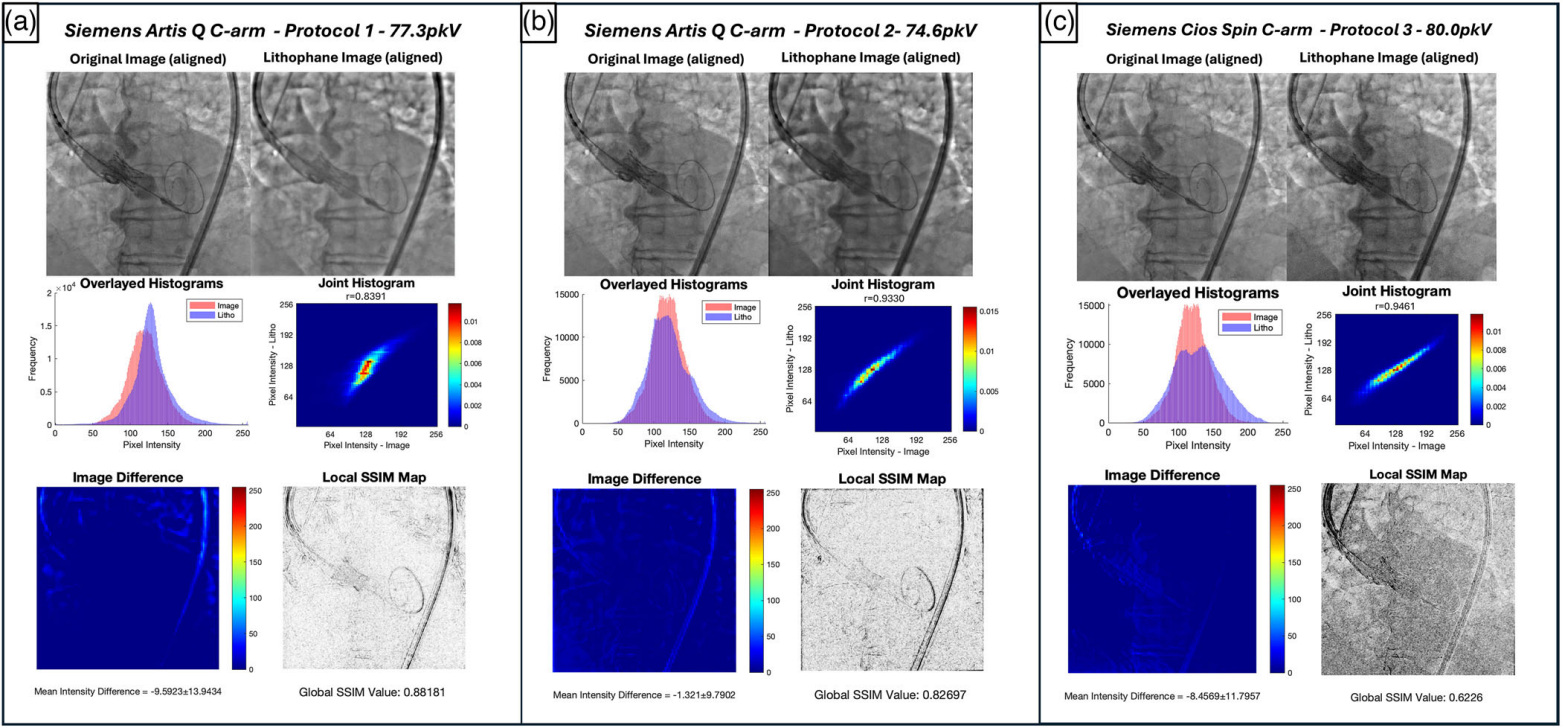
This figure shows a direct comparison between the original image from the patient, and the lithophane images acquired with the Artis Q and Cios Spin x‐ray systems. The images were numerically compared using the histograms, 2D joint histogram, intensity difference, and SSI.

### 3.3 AI‐based evaluation

All fluoroscopy images from Simulations 1 and 2 were input to the FDA cleared (510(k))TAVIPILOT software post‐operatively. In the lithophane images, all landmarks were identified and marked correctly both on the lithophane in Simulation 1 and during valve alignment/deployment in Simulation 2. Though all dots were placed on the correct features in all images, there were minor (<1 mm) translations and differences visible in the locations of the dots between images acquired with protocols 1, 2, and 3. The TAVIPILOT outputs for Simulation 1 and Simulation 2 can be seen in Figure 9; blue, green, pink and purple dots mark the implant, and red dots mark the pigtail catheter. Like in patients, the AI effectively tracked all landmarks during alignment and focused on the implant during deployment, as shown in the second and third rows of Figure 9.

**FIGURE 9:**
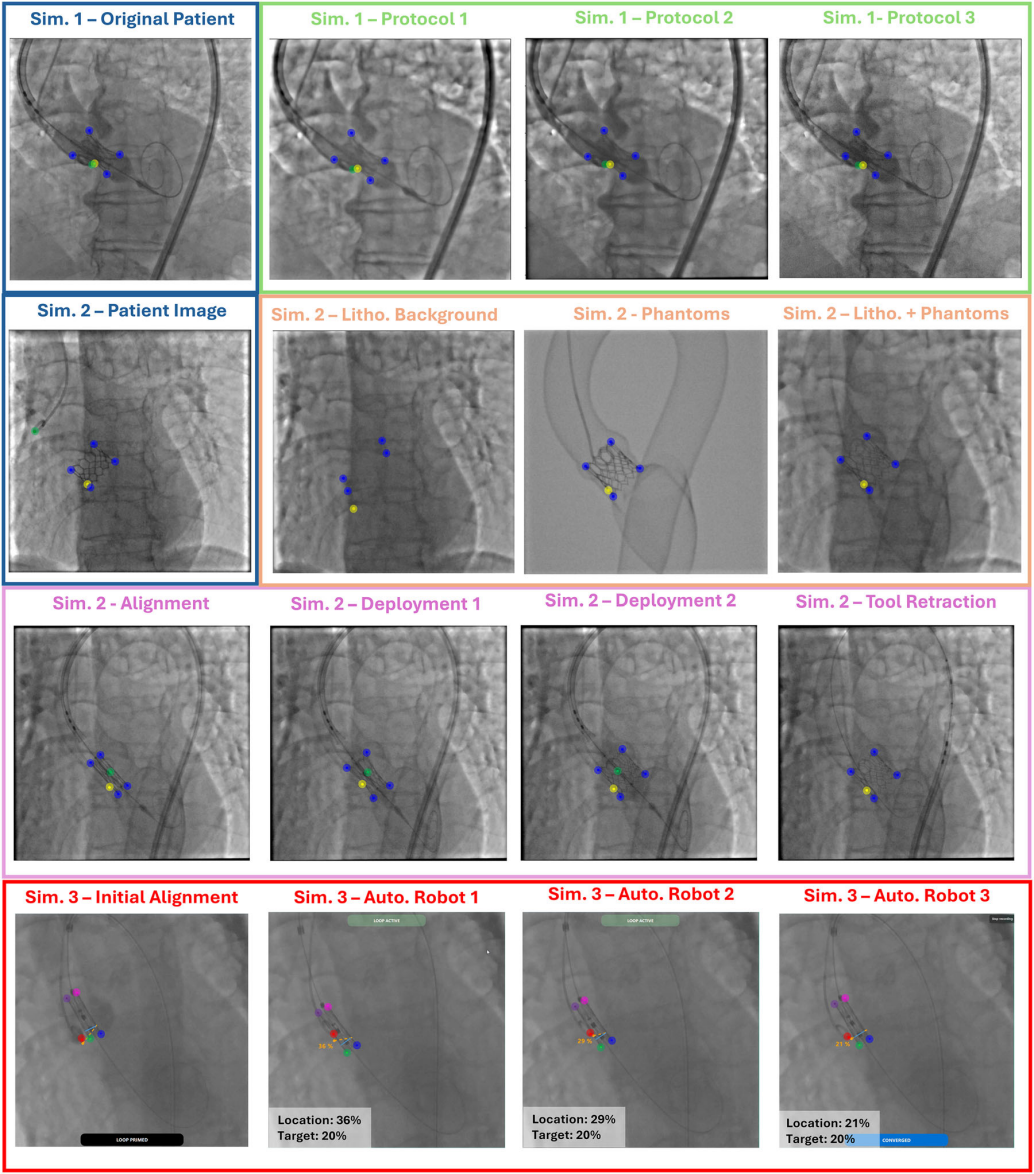
This figure shows the TAVIPILOT landmark detection in: the original patient (blue); the lithophane images from Simulation 1 (green); the various layers of simulation technologies used in Simulation 2 (orange); the dynamic sequences of Simulation 2 (pink). The bottom row (red) shows the TAVIPILOT output during autonomous AI‐driven robotic guidance until the stent phantom's location coincided with the target location in the aortic valve. The robot was active throughout the loop sequence.

Simulation 3 resulted in a successful alignment of the stent phantom using the Caranx Medical robot autonomously driven by TAVIPILOT. The software was able to detect and track all points of the valve, pigtail catheter and non‐coronary cusp as they were moved in the beating heart and contrast agent was injected. The software treated the phantom as a patient, maintained landmark detection throughout the procedure, and autonomously guided the implant to its desired location. The TAVIPILOT output for Simulations 1, 2, and 3 can be observed Figure 9.

### 3.4 Survey‐based evaluation

In the first part of the survey, the participants were able to spot the fakes and answer correctly 36.00% ± 12.94% of the times. In a two tailed t‐test at 5% significance there was no statistically significant difference between the scores and random selection at 1/4 (25%) (*p* = 0.1302). Table 1 displays the average rating for each field among the volunteers and the average rating weighed by their respective scores to the first part of the questionnaire. There is no statistically significant difference between the average Likert scores and the average Likert scores weighted by participant accuracy (Table 1). Two final comments were recorded: “Good work” and “The fake pictures are very realistic for fluoroscopy.”

**TABLE 1:** Questionnaire per‐question average scores, weighted average scores (± SD), and the results of a two‐tailed *t*‐test (all showing no significant difference between averages and weighted averages).

Question	Mean ± SD	W. Mean ± SD	*t*‐test
Image sharpness	3.32 ± 0.46	3.38 ± 0.41	*p* = 0.7822
Fidelity to clinical appearance	3.56 ± 0.65	3.56 ± 0.60	*p* = 0.9891
Grey‐scale contrast	3.48 ± 0.36	3.48 ± 0.35	*p* = 0.9853
Noise management	3.24 ± 0.67	3.28 ± 0.63	*p* = 0.9098
Visibility of critical details	3.56 ± 0.22	3.57 ± 0.22	*p* = 0.9515
Overall realism / diagnostic usability	3.48 ± 0.23	3.43 ± 0.17	*p* = 0.6445

## 4 DISCUSSION

Figures 8 and 9 clearly show how lithophanes were effective in replicating the human body and its appearance in fluoroscopy, as well as enhancing existing simulators and phantoms. Unlike in N. Irnstorfer et al.,^26^ which used 7.5 cm of plastic to simulate pediatric patients, this method was able to replicate adult patients using only up to 3 cm of plastic. This simplification in manufacturing expands the potential uses for this technology. The images were able to recreate even the finer details of the image, including the guide‐wires and the stent of the implant. To render the methods more accessible to the wider community, our methods printed small lithophanes on a popular consumer‐grade 3D‐printer with a standard 0.4 mm hardened steel nozzle. This manufacturing modality was able to produce ready‐to‐use lithophane with no visible manufacturing defects, and requiring no post processing procedures. All the catheters and guidewires used in these source images for the lithophanes were larger than 0.4 mm and could thus be effectively replicated by the printer; to replicate even finer interventional equipment with higher detail, the lithophanes could be printed larger than life‐size, with a thinner layer height (<0.2 mm), using a finer nozzle (<0.4 mm), minimizing the effects of manufacturing resolution. A more accurate but costly solution may be to adopt a different manufacturing modality instead, such as industrial powder‐based 3D‐printing, to improve manufacturing resolution (∼0.04 mm accuracy^50^). Our methods made use of MATLAB code to create the lithophane STLs however, this could be alternatively achieved using open‐source 3D‐printing slicer Cura (Ultimaker, Utrecht, Netherlands) by importing the images as PNG or TIFF files and adjusting the dimensions in the menu.

In the software, mathematical model 1 allowed for quick and reliable (*r* = 0.933, *p* < 0.0001) estimation of the thickness necessary for each lithophane. Images with 8‐bit minimum intensities between 37 and 152 can be replicated with lithophanes with thickness 1–30 mm. The model 1 function will plateau towards minimum pixel intensity ∼31, as thickness tends to infinity. Because of this, a 30 mm thickness limit was imposed in our methods. Future studies could make use of more attenuative materials, such as 3D‐printed steel or copper, to accommodate for intensities lower than 30 and achieve a wider intensity range of values. All lithophanes were set to have a minimum thickness of 0.8 mm. This was done to maintain structural integrity and to avoid holes in the brightest regions of the lithophane. The maximum achievable intensity allocated to these thinnest areas was estimated based on mathematical model 2. The function accurately followed the linear path set by the datapoints (*r* = 0.953, *p* < 0.0001), and its implementation maintained the lithophanes' structural integrity whilst still achieving a realistic radiographic appearance. These estimated functions may vary between different x‐ray machines and different imaging protocols. Future studies could present more universal functions derived using multiple x‐ray systems from different manufacturers using 16‐bit pixel intensities to minimize sampling error.

The numerical analysis of Simulation 1 suggests that the lithophane closely resembled the original patient image on both x‐ray systems. Protocol 1 achieved a similar peak energy (77.3 pkV) to the original image (76.8 pkV). The lithophane image appear blurrier (VoL = 2) than the original (VoL = 37), which may have ultimately reduced the linearity of the joint histogram and increased the NRMSE (0.0656), however, the mean intensities and standard deviations overlapped, and the global SSI value was relatively high (0.882), indicating that the two images were still very similar. Using Protocol 2 (74.6 pkV) improved the joint histograms (*r* = 0.933), image sharpness (VoL = 21), NRMSE (0.0393), and the pixel difference (‐1±10); however the global SSI value was worsened (0.827). Dark artefacts on the borders of the local SSI map indicate that horizontal misalignment may have effected the global SSI value. This protocol produced the lowest NRMSE (<0.05), indicating that this image may arguably be the closest match the original patient's. Protocol 3 also achieved a similar peak energy (80.0 pkV) to the original patient image. The lithophane image had a sharper appearance (VoL = 354) than the original, but was arguably affected by more noise than the other two (global SSI = 0.623).

Across all three lithophane images form Simulation 1, the intensity difference and local SSI maps around the guide‐wires suggest that misalignment may have affected the similarity metrics: more accurate results may be achieved by implementing different iterative alignment functions either manually or automatically.

The patterns of the noise on the maps closely resemble the surface of the lithophane, suggesting these artifacts may have been related to the 3D‐printing process. Despite this, the VoL measurements from Simulation 1 show that the current printing method can preserve image sharpness comparable to the original image when similar fluoroscopy settings are used. Mitigating image noise to create a smoother lithophane like in N. Irnstorfer et al.^26^ may solve this minor flaw, however it introduces the possibility of losing textural features or blurring the image.

The joint histogram between the Cios‐Spin x‐ray system's image and the original show a highest level of linearity (r>0.95) and maintains a relatively low NRMSE (0.0599), indicating that these x‐ray settings and intensity range may more closely resemble those of the original image. The intensity difference and local SSI map suggest there may have been minor misalignment around the aortic root, however, speckled noise in the SSI map suggests most of the differences in this image may arise from the physical surface texture of the lithophane. In addition to this manufacturing shortcoming, the lithophanes were created from real x‐ray images which natively include noise; this native noise from the original image is therefore included in the design of the printed lithophane. Further noise is then added when imaging the lithophane, so the final image inherently contains more noise. A similar summative effect also occurs with spacial resolution: the original image contains some blurring from the spacial resolution of the original fluoroscopy acquisition, which is reflected on the lithophane design and then repeated when imaging the lithophane. The methods employed in this study made sure to minimize the impact of these optical effects by using high‐resolution original images to craft the lithophanes.

Despite limitations in image alignment, surface texture, and optical effects, Simulation 1 proved x‐ray lithophanes can realistically replicate patient images. Due to the level of realism achieved, the TAVIPILOT output from Simulation 1 images shows landmarks being detected as if all images were acquired from patients. This proves that lithophanes could find applications in testing and evaluating AI software. With relatively low costs, lithophanes such as these can promote rapid prototyping and iterative engineering in radiology without exposing patients or operators to more radiation. Unlike software‐based simulators, lithophanes were directly deployed in the catheterization laboratory without needing to modify the control room or download new programs. The main limitation to using only single lithophane test plates lies in their static nature, which prevents them from replicating dynamic scenes on their own.

Simulations 2 and 3 introduced movement to lithophane simulation, using rigid and flexible phantoms both in air and in water. The TAVIPILOT system detected all landmarks and tracked them dynamically, enabling it to autonomously drive Caranx Medical's robotic system, treating the simulator as a patient. In Simulation 2 and 3, the physical stents and guide wires appeared sharper than in Simulation 1, highlighting the previously discussed manufacturing limitations when including fine interventional tools directly in the lithophane's source image. As highlighted by the second row of Figure 9, pairing the aortic phantom with the lithophane background resolved the static nature of Simulation 1 while also adding a layer of radiographic realism otherwise unachievable with 3D‐printed organ phantoms. Submerging and perfusing the vascular phantoms in water allows users to simulate pumping blood and the surrounding soft tissue. As water, soft tissues, and plastics exhibit similar x‐ray properties, submerging the plastic/rubber aortas in water also hides them in fluoroscopy. Though still effective, Figure 9 shows that adding the water tank in Simulation 3 can affect the visibility of the lithophane by making the lithophane appear more homogeneously grey in color, thus reducing contrast. This may be due to the water being more attenuating than air, and thus, reducing the relative difference in photons being detected in different areas of the lithophane. In order to compensate this effect in water, future works could repeat our methods to recalibrate the mathematical functions in water employing different x‐ray settings or thicker lithophanes made with more radiopaque materials to compensate for water's radiopacity.

The survey‐based evaluation reinforces what was shown by the previous evaluations. The IHU Strasbourg research cath‐labs provided the ideal location to recruit volunteers with experience (up to 30 years in their current role) and high exposure (1–20+ cases per week) to cardiovascular fluoroscopy. Despite their expertise, the participants accuracy (36.00% ± 12.94%) did not significantly differ from random selection (*p* = 0.1302). Arguably, this indicates that lithophanes can look realistic among other similar fluoroscopy images. This was also mirrored in the two comments recorded in the study, praising the “work” and the realism of the lithophanes “for fluoroscopy.” Answers to the second part of the survey showed that the lithophane not only maintain resemblance of their original image, as proven in Simulation 1, but also score “Adequate” or “Good” (3.24–3.56) on all fields of clinical appearance. When weighting the scores based on the individual's accuracy, no significant difference was observed from the overall average, meaning the results to the Likert questions were independent of the volunteer's proficiency in distinguishing pairs.

The survey responses, mathematical evaluation, and outputs from TAVIPILOT all show that lithophanes can reliably recreate patient images and enhance the radiographic appearance of interventional phantoms, providing highly‐realistic uniplanar simulation environment to test AI systems such as TAVIPILOT. The same principle could also be applied in the training of new operators. Future studies may investigate the benefits associated to more realistic x‐ray appearance during simulation‐based staff training sessions for TAVI as well as other cardiovascular interventional procedures.

Physical lithophanes and cardiovascular simulators offered a patient‐like, quick‐to‐deploy solution ideal for controlled live testing in the catheterization laboratory using clinical equipment exactly as it is used in patient practice. This approach is best suited to industrial validation and regulatory testing (e.g. FDA, CE) and clinical sites where users cannot modify their x‐ray systems, because it does not impose changes to the acquisition hardware or software, thus preserving the device's native output. By contrast, live digital overlays and augmented reality approaches may reproduce comparable results but would demand additional preparation, equipment, and expertise, and may introduce workflow disruptions and additional time delays between the ground truth and the displayed image. For these reasons our approached focused on purely physical simulation for rapid, repeatable testing. Future studies should investigate the differences between deploying radiographic lithophanes and live digital overlays for device and software validation.

## 5 CONCLUSIONS

This study shows that, x‐ray lithophanes constitute a cost‐effective and realistic replacement for human subjects, cadavers or animals in radiology applications, enabling for reliable repeatable tests whilst avoiding unnecessary exposure to radiation. This technology can be used to streamline test protocols and limit the need of staff or patients in contact with radiation during experiments. Simulation 1 shows how single fluoroscopic shots can be replicated, allowing users to prepare a range of patient‐like test images that can be carried and deployed at the point of care for quick repeatable software tests. Simulation 2 and 3 showed how this technology can be paired with 3D organ phantoms to achieve a more realistic x‐ray appearance during simulation. These simulations incorporated moving objects as well as a hyper‐realistic background, without the need for more sophisticated 3D radiographic phantoms. When repeatedly testing AI for clinical use, such phantoms allow users to deploy and test new software in the cath‐lab on human‐like anatomy without resorting to volunteers, virtual reality augmentation post‐acquisition, or specialized cadaver facilities. This proof‐of‐concept study opens the door to new research, applying this same lithophane principle to other fields of radiology, software development, and staff training.

## CONFLICT OF INTEREST STATEMENT

Authors Carlo Saija, Federica Facente, Wen Wei, Eric Sejour and Pierre Berthet‐Ryne were employed by the company Caranx Medical. The remaining authors declare that this research was conducted in the absence of any commercial or financial relationships that could be construed as potential conflicts of interest.

## FUNDING INFORMATION

This work was supported by the Wellcome Centre for Medical Engineering at King's College London [WT203148/Z/16/Z]; the British Heart Foundation (BHF) Centre of Excellence at King's College London; the Department of Health and Social Care (DHSC) through the National Institute for Health and Care Research (NIHR) MedTech; and Vitro Diagnostic Co‐operative (MIC) Award for Cardiovascular Diseases to Guy's & St Thomas' NHS Foundation Trust in partnership with King's College London [MIC‐2016‐019]. This work was supported by funding from Caranx Medical, Nice, France.

## ETHICAL APPROVAL

Ethical approval for the volunteer‐based evaluation was obtained from the King's College London ethics committee (MRSP‐24/25‐51316). To preserve participant anonymity, volunteers were provided with a full description of the study before participation, and completion of the anonymous questionnaire was taken as implied informed consent. No personally identifiable or traceable information was collected.

## Data Availability

The data that support the findings of this study are available from the corresponding author upon reasonable request.

## References

[1] E. S. of Radiology . The future role of radiology in healthcare. Insights Imaging. 2010;1:2–11. 10.1007/s13244-009-0007-x PMC3259353 22347897

[2] Kwee TC , Almaghrabi MT , Kwee RM . Diagnostic radiology and its future: what do clinicians need and think?. Eur Radiol. 2023;33:9401‐9410. 10.1007/s00330-023-09897-2 PMC10667510 37436504

[3] Lanza C , Carriero S , Buijs EFM , et al. Robotics in interventional radiology: review of current and future applications. Technol Cancer Res Treat. 2023;22:15330338231152084. 10.1177/15330338231152084 PMC10150437 37113061

[4] Schmidgall S , Opfermann JD , Kim JW , Krieger A . Will your next surgeon be a robot? Autonomy and AI in robotic surgery. Sci Robot. 2025;10:eadt0187. 10.1126/scirobotics.adt0187 PMC12836364 40700524

[5] Denolf PL , Vanderveken OM , Marklund M , Braem M . The status of cephalometry in the prediction of non‐CPAP treatment outcome in obstructive sleep apnea patients. Sleep Med Rev. 2016;27:56‐73. 10.1016/j.smrv.2015.05.009 26452001

[6] Wang J , Blackburn TJ . The AAPM/RSNA physics tutorial for residents. Radiographics. 2000;20:1471‐1477. 10.1148/radiographics.20.5.g00se181471 10992034

[7] Livyatan H , Yaniv Z , Joskowicz L . Robust automatic C‐Arm calibration for fluoroscopy‐based navigation: a practical approach. In Proceedings of MICCAI 2002 , MICCAI 2002, San Francisco. Springer Science+Business Media; 2002. doi: 10.1007/3-540-45787-9_8

[8] Tate PM , Lachine V , Fu L , Croitoru H , Sati M . Performance and robustness of automatic fluoroscopic image calibration in a new computer assisted surgery system. In Proceedings of MICCAI 2001 , MICCAI 2001, San Francisco. Springer Science+Business Media; 2001. doi: 10.1007/3-540-45468-3_135

[9] Gustav H , Jørgensen TH , Ihlemann N , et al. Transcatheter or surgical aortic valve implantation: 10‐year outcomes of the NOTION trial. Eur Heart J. 2024;45:1116‐1124. 10.1093/eurheartj/ehae043 PMC10984572 38321820

[10] Bourantas CV , Modolo R , Baumbach A , et al. The evolution of device technology in transcatheter aortic valve implantation. Eurointervention. 2019;14:e1826–e1833. 10.4244/EIJ-D-18-01048 30719977

[11] Osnabrugge RLJ , Mylotte D , Head SJ , et al. Aortic stenosis in the elderly. J Am Coll Cardiol. 2013;62:1002‐1012. 10.1016/j.jacc.2013.05.015 23727214

[12] Bleiziffer S , Appleby C , Delgado V , et al. Patterns of aortic valve replacement in Europe and adoption by sex. Int J Cardiol. 2024;406:131996. 10.1016/j.ijcard.2024.131996 38555056

[13] Kalra A , Reardon M , Barker C , Kleiman N , Reyes M . Transcatheter Heart Valve Handbook: A Surgeons' and Interventional Council Review. American College of Cardiology; 2015.

[14] Fortuni F , Ciliberti G , De Chiara B , et al. Advancements and applications of artificial intelligence in cardiovascular imaging: a comprehensive review. Eur Heart J Imaging Methods Pract. 2024;2:qyae136. 10.1093/ehjimp/qyae136 PMC11705385 39776818

[15] TaviPilot Solution — Caranx Medical, Online, 2025, Accessed October 7, 2025. https://caranx‐medical.com/our‐solution/tavipilot‐solution/

[16] Caranx Medical, Online, 2025, Accessed October 7, 2025. https://caranx‐medical.com/

[17] Smits J , Schegg P , Wauters L , et al. Towards autonomous robot‐assisted transcatheter heart valve implantation. Front Rob AI. 2025;12:1650228. 10.3389/frobt.2025.1650228 PMC12583050 41194941

[18] Collier R . Switching swine for simulators: at what cost?. Can Med Assoc J. 2008;179:759‐760. 10.1503/cmaj.081400 PMC2553857 18838446

[19] DiVincenti L , Westcott R , Lee C . Sheep (*Ovis aries*) as a model for cardiovascular surgery and management before, during, and after cardiopulmonary bypass. J Am Assoc Lab Anim Sci. 2014;53:439. PMC4181684 25255065

[20] Lustgarten DL , Bell S , Hardin N , Calame J , Spector PS . Safety and efficacy of epicardial cryoablation in a canine model. Heart Rhythm. 2005;2:82‐90. 10.1016/j.hrthm.2004.09.022 15851270

[21] Carey JN , Minneti M , Leland HA , Demetriades D , Talving P . Perfused fresh cadavers: method for application to surgical simulation. Am J Surg. 2015;210:179‐187. 10.1016/j.amjsurg.2014.10.027 25890815

[22] Kovacs G , Levitan R , Sandeski R . Clinical cadavers as a simulation resource for procedural learning. AEM Educ Train. 2018;2:239‐247. 10.1002/aet2.10103 PMC6050063 30051097

[23] Przybylski F , Adagolodjo Y , Dequidt J , Duriez C , Berthet‐Rayne P . Enhancing TAVI robot flexibility using semi‐extensible materials. Presented at HSMR 2024‐16th Hamlyn Symposium on Medical Robotics , 2024, Accessed February 4, 2025. https://hal.science/hal‐04646160

[24] Wang W , Wu Z , Saija C , et al. Universal actuation module and kinematic model for heart valve interventional catheter robotization. IEEE Robot Autom Lett. 2024;9:5254‐5261. 10.1109/LRA.2024.3388789 PMC7618158 40989075

[25] Mille MM , Griffin K , Maass‐Moreno R , Lee C . Fabrication of a pediatric torso phantom with multiple tissues represented using a dual nozzle thermoplastic 3D printer. J Appl Clin Med Phys. 2020;21:226‐236. 10.1002/acm2.13064 PMC7701110 33073922

[26] Irnstorfer N , Unger E , Hojreh A , Homolka P . An anthropomorphic phantom representing a prematurely born neonate for digital x‐ray imaging using 3D printing: Proof of concept and comparison of image quality from different systems. Sci Rep. 2019;9:14357. 10.1038/s41598-019-50925-3 PMC6779877 31591433

[27] Wang W , Wu Z , Saija C , et al. Universal actuation module and kinematic model for heart valve interventional catheter robotization. IEEE Robotics and Automation Letters. 2024;9:5254‐5261. 10.1109/LRA.2024.3388789 PMC7618158 40989075

[28] Torabinia M , Caprio A , Jang S‐J , et al. Deep learning‐driven catheter tracking from bi‐plane X‐ray fluoroscopy of 3D printed heart phantoms. Mini‐invasive Surg. 2021;5:32.

[29] Jianu T , Huang B , Nguyen H , et al. Guide3D: a bi‐planar x‐ray dataset for 3D shape reconstruction. ACCV 2024. 2024;1:366‐382.

[30] Jianu T , Huang B , Berthet‐Rayne P , Fichera S , Nguyen A . 3D guidewire shape reconstruction from monoplane fluoroscopic images. RiTA. 2023 2024;1:84‐94.

[31] Chen F , Chen H , Yu T , et al. AI‐driven revolution of medical robotics across surgical innovation, rehabilitation intelligence, and multimodal healthcare delivery. MedComm. 2026;7:e70597. 10.1002/mco2.70597 PMC12968330 41810390

[32] Gennaro G , Zanardo M , Ambrogi F , Sardanelli F . Phantom studies in medical imaging (PSMI): a guide with recommendations and checklist. Eur Radiol Exp. 2025;9:105. 10.1186/s41747-025-00641-7 PMC12583249 41182488

[33] Smits J , Schegg P , Wauters L , et al. Towards autonomous robot‐assisted transcatheter heart valve implantation: in vivo teleoperation and phantom validation of AI‐guided positioning. Front Rob AI. 2025;12:1650228. 10.3389/frobt.2025.1650228 PMC12583050 41194941

[34] Saija C , Sabu S , Leung L , et al. Evaluation of a three‐dimensional printed interventional simulator for cardiac ablation therapy training. Appl Sci. 2024;14:8423.

[35] Torabinia M , Caprio A , Jang S‐J , et al. Deep learning‐driven catheter tracking from bi‐plane X‐ray fluoroscopy of 3D printed heart phantoms. Mini‐invasive Surg. 2021;5:32.

[36] Walsh K , Jaye P . The relationship between fidelity and cost in simulation. Med Educ. 2012;46:1226. 10.1111/j.1365-2923.2012.04352.x 23171265

[37] Bettati P , Dormer JD , Young J , Shahedi M , Fei B . Virtual reality assisted cardiac catheterization. Proceedings of SPIE – The International Society for Optical Engineering. 2021;11598:115982D. 10.1117/12.2582097 PMC8849600 35177877

[38] Hecko J , Precek D , Januska J , et al. Design and validation of a mixed reality workflow for structural cardiac procedures in interventional cardiology. Front Virtual Real 2025;6:1690439.

[39] Chahal B , Aydin A , Ahmed K . Virtual reality vs. physical models in surgical skills training. An update of the evidence. Curr Opin Urol. 2024;34:32‐36. 10.1097/MOU.0000000000001145 PMC10715699 37962178

[40] Aslani N , Behmanesh A , Garavand A , Maleki M , Davoodi F , Shams R . The virtual reality technology effects and features in cardiology interventions training: a scoping review. Med J Islam Repub Iran. 2022;36:77. 10.47176/mjiri.36.77 PMC9448494 36128285

[41] Berger M , Yang Q , Maier A . X‐ray Imaging: An Introductory Guide . In Maier A, Steidl S, Christlein V, Hornegger J, editor. Medical Imaging Systems: Lecture Notes in Computer Science.*Springer Open*; 2018 p.119‐138. 10.1007/978-3-319-96520-8_7

[42] Das I , Cheng C‐W , Cao M , Johnstone PAS . Computed tomography imaging parameters for inhomogeneity correction in radiation treatment planning. J Med Phys. 2016;41:3. 10.4103/0971-6203.177277 PMC4795414 27051164

[43] Bolliger SA , Oesterhelweg L , Spendlove D , Ross S , Thali MJ . Is differentiation of frequently encountered foreign bodies in corpses possible by Hounsfield density measurement?. J Forensic Sci. 2009;54:1119‐1122. 10.1111/j.1556-4029.2009.01100.x 19627414

[44] Ma X , Figl M , Unger E , Buschmann M , Homolka P . X‐ray attenuation of bone, soft and adipose tissue in CT from 70 to 140 kV and comparison with 3D printable additive manufacturing materials. Sci Rep. 2022;12:14580. 10.1038/s41598-022-18741-4 PMC9418162 36028638

[45] Qanadli SD , Jouannic A‐M , Dehmeshki J , Lu T‐L . CT attenuation values of blood and myocardium: rationale for accurate coronary artery calcifications detection with multi‐detector CT. PLoS One 2015;10:e0124175. 10.1371/journal.pone.0124175 PMC4397043 25875629

[46] Safety data sheet ‐ composite iron fiber PLA, Online resource, 2018, Accessed February 4, 2025. https://cdn.shopify.com/s/files/1/0717/9095/files/FEP1xxxx_SDS.pdf?1992606272897634343

[47] Arshi A , Yakubov SJ , Stiver KL , Sanchez CE . Overcoming the transcatheter aortic valve replacement Achilles heel: coronary re‐access. Ann Cardiothorac Surg. 2020;9:468‐477. 10.21037/acs-2020-av-38 PMC7724072 33312904

[48] Pollard BJ , Samei E , Chawla AS , Delong DM , Yoshizumi TT . The effects of ambient lighting in chest radiology reading rooms. J Digit Imaging. 2012;25:520‐526. 10.1007/s10278-012-9459-5 PMC3389094 22349990

[49] Butler ML , Rainford LA , Brennan PC . The effect of viewing distance on observer performance in skeletal radiographs. J Digit Imaging. 2013;26:653‐660.

[50] Seltzman AH , Wukitch SJ . Resolution and geometric limitations in laser powder bed fusion additively manufactured GRCop‐84 structures for a lower hybrid current drive launcher. Fusion Eng Des. 2021;173:112847.

